# DDX3X and DDX3Y are redundant in protein synthesis

**DOI:** 10.1261/rna.078926.121

**Published:** 2021-12

**Authors:** Srivats Venkataramanan, Margaret Gadek, Lorenzo Calviello, Kevin Wilkins, Stephen N. Floor

**Affiliations:** 1 Department of Cell and Tissue Biology, University of California, San Francisco, San Francisco, California 94143, USA; 2 Helen Diller Family Comprehensive Cancer Center, University of California, San Francisco, San Francisco, California 94143, USA

**Keywords:** translational control, DEAD-box proteins, RNA, sex differences

## Abstract

DDX3 is a DEAD-box RNA helicase that regulates translation and is encoded by the X- and Y-linked paralogs *DDX3X* and *DDX3Y*. While DDX3X is ubiquitously expressed in human tissues and essential for viability, DDX3Y is male-specific and shows lower and more variable expression than DDX3X in somatic tissues. Heterozygous genetic lesions in *DDX3X* mediate a class of developmental disorders called DDX3X syndrome, while loss of *DDX3Y* is implicated in male infertility. One possible explanation for female-bias in DDX3X syndrome is that *DDX3Y* encodes a polypeptide with different biochemical activity. In this study, we use ribosome profiling and in vitro translation to demonstrate that the X- and Y-linked paralogs of DDX3 play functionally redundant roles in translation. We find that transcripts that are sensitive to DDX3X depletion or mutation are rescued by complementation with DDX3Y. Our data indicate that DDX3X and DDX3Y proteins can functionally complement each other in the context of mRNA translation in human cells. DDX3Y is not expressed in a large fraction of the central nervous system. These findings suggest that expression differences, not differences in paralog-dependent protein synthesis, underlie the sex-bias of DDX3X-associated diseases.

## INTRODUCTION

DDX3X is a ubiquitously expressed ATPase and RNA-helicase encoded by an essential gene on the X-chromosome, *DDX3X*. *DDX3X* escapes X-inactivation in a wide range of tissues (Cotton et al. 2015). Mutations in *DDX3X* are associated with numerous pathologies, including cancers like medulloblastoma (Kool et al. 2014; Floor et al. 2016; Oh et al. 2016), chronic lymphocytic leukemia (Ojha et al. 2015), squamous cell carcinoma (Stransky et al. 2011), Burkitt's lymphoma (Gong et al. 2021), and many others (Sharma and Jankowsky 2014). Heterozygous missense or loss-of-function mutations in *DDX3X* are also implicated in intellectual disability and autism-spectrum disorders in females (Iossifov et al. 2014; Snijders Blok et al. 2015; Yuen et al. 2017; Takata et al. 2018; Wang et al. 2018; Ruzzo et al. 2019; Scala et al. 2019; Lennox et al. 2020), with the severity of phenotype correlating with the degree of reduction in DDX3X catalytic activity (Lennox et al. 2020).

*DDX3X* has a Y-chromosome paralog, *DDX3Y*, and the protein products of these two genes are more than 90% identical (Ditton et al. 2004). *DDX3Y* is located within the *Azoospermia Factor a (AZFa)* locus nested within the nonrecombining region of the Y chromosome (Kotov et al. 2017). Expression of *DDX3Y* alone has been shown to rescue the specific infertility phenotype caused by deletion of *AZFa* (Ramathal et al. 2015), although these findings have been disputed in mice (Matsumura et al. 2019). *DDX3Y* mRNA is broadly expressed across tissues, but activation of a testis-specific distal promoter produces a transcript isoform with a distinct 5′ leader that also contributes to its translational control (Jaroszynski et al. 2011). Because of this, expression of DDX3Y was thought to be testis-specific, but later work suggested that DDX3Y is expressed more broadly in tissues across the human body (Uhlen et al. 2015) (data available from v19.proteinatlas.org). Furthermore, recent evidence suggests that depletion of DDX3Y results in neural differentiation defects, indicating wider tissue distribution of DDX3Y than previously assumed (Vakilian et al. 2015). Notably, *DDX3Y* expression is consistently lower than that of its X-linked paralog (Uhlen et al. 2015) (data available from v19.proteinatlas.org).

*DDX3X* and *DDX3Y* both encode for similar DDX3 polypeptides, a DEAD-box RNA chaperone that facilitates translation initiation on mRNAs with structured 5′ leaders (Oh et al. 2016; Calviello et al. 2021). We recently defined the set of genes that depend on DDX3X for their translation and proposed a model where this translation is facilitated by the resolution of these highly structured 5′ leaders by 40S-associated DDX3X (Calviello et al. 2021), consistent with prior work (Chen et al. 2018). However, the role of DDX3Y in translation and gene expression—and how similar or different its function is to DDX3X—remains incompletely understood.

Genetic diseases involving *DDX3X* such as DDX3X syndrome demonstrate severe sex bias. A recent cohort (*n* = 107, 104 females, three males) contains de novo genetic lesions in *DDX3X* that are exclusively heterozygous (or hemizygous in males) (Lennox et al. 2020). These observations have been attributed to the inability of the Y-linked DDX3 paralog to functionally complement *DDX3X*, leading to embryonic lethality in males with inactivating mutations of *DDX3X*—whereas heterozygous females are viable, but suffer from symptoms of varying intensity. *Ddx3y* is unable to compensate for the loss of *Ddx3x* during embryonic development in mice (Chen et al. 2016). This is in contrast to the observation that mouse *Ddx3y i*s able to compensate for the loss of *Ddx3x* during neural development, insulating male mice from ataxia and seizure phenotypes upon ablation of *Ddx3x*, but is unable to reverse the susceptibility to hindbrain malignancies conferred by *Ddx3x* loss (Patmore et al. 2020). Conversely, the loss of *DDX3Y* confers infertility in human males, despite the robust expression of *DDX3X* in germ-line tissues (Ramathal et al. 2015). In principle, sex bias in DDX3X-dependent disorders could arise from differential expression between *DDX3X* and *DDX3Y* across tissues, different activities of their gene products, dosage compensation by the wild-type locus, or other unknown mechanisms.

In this study, we tested the functions of DDX3X and DDX3Y proteins in translation through cell-based and biochemical assays. We depleted endogenous DDX3X protein in male HCT 116 cells using an inducible degron system, complemented with either *DDX3X* or *DDX3Y* cDNAs, and measured translation and RNA abundance using ribosome profiling and RNA-seq. We find that *DDX3Y* or *DDX3X* complements DDX3X loss in protein synthesis. Using an in vitro reporter system, we demonstrate that genes that are robustly susceptible to depletion of DDX3 do not show significant changes upon substitution of DDX3X with DDX3Y. We further find that DDX3X and DDX3Y proteins have similar stability in cells. Taken together with their distinct tissue-level expression patterns, our data suggest that the X- and Y-linked paralogs of DDX3 are redundant in protein synthesis, implying tissue-specific variation of DDX3Y expression or another mechanism beyond translational control underlies the sex bias of DDX3X-associated developmental disorders.

## RESULTS

**FIGURE 1.**
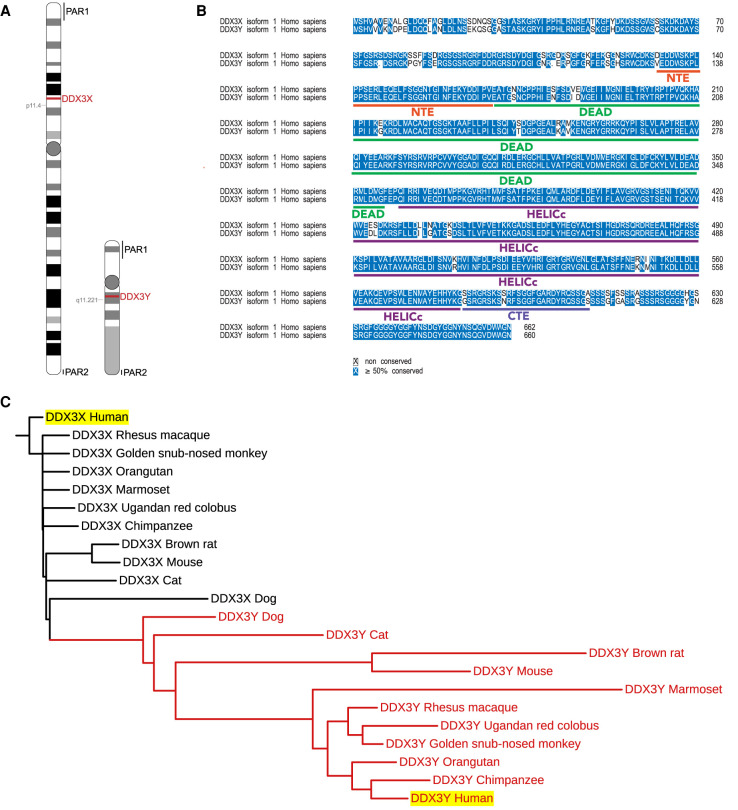
(*A*) Graphic indicating the locations of *DDX3X* and *DDX3Y* on their respective chromosomes. Pseudoautosomal regions (PAR) are indicated. (*B*) Alignment of human DDX3X and DDX3Y demonstrating ∼92% sequence identity. Domain architecture of DDX3X is indicated. (*C*) Phylogenetic tree indicating distances between the sequences of DDX3X and DDX3Y in mammals (where sequence is available, only selected species indicated, see Supplemental Figure S1 for tree of all available mammalian sequences). The cluster of mammalian DDX3Y orthologs is indicated with red branches. Human orthologs are highlighted in yellow.

The DEAD-box RNA helicase DDX3 has two sex-linked paralogs. The X-linked *DDX3X* gene is located on a nonpseudoautosomal region of the short arm of the X-chromosome (Fig. 1A). *DDX3Y*, a paralog of *DDX3X*, is located on the long arm of the Y chromosome in the male-specific region (Fig. 1A). The sex bias of DDX3X syndrome suggests that males with inactivating mutations in DDX3X suffer embryonic lethality, implying that human *DDX3Y* cannot functionally complement *DDX3X*. However, *DDX3X* and *DDX3Y* protein products show ∼92% homology, with most of the differences in the amino-terminal domain (Fig. 1B), which is dispensable for RNA duplex unwinding in vitro (Floor et al. 2016). In contrast, the amino-terminal domain of DDX3 is important for protein–protein interactions and contains a nuclear export sequence (Shih et al. 2012; Floor et al. 2016), suggesting that changes to this region in DDX3X versus DDX3Y proteins could result in functional changes in cells. A phylogenetic tree of available mammalian DDX3 protein sequences reveals significant homology between mammalian DDX3Y, and separation of the “DDX3Y clade” away from DDX3X (Fig. 1C; Supplemental Fig. S1). In many cases, DDX3Y sequences of evolutionary distant mammals show greater homology than the paralogs within a given species (Fig. 1C; Supplemental Fig. S1). However, this phylogenetic tree also suggests that DDX3X proteins are more highly conserved than DDX3Y proteins, consistent with comparatively fewer missense variants in DDX3X compared to DDX3Y in humans in the gnomAD data set (missense *Z* score of 2.1 for DDX3Y vs. 4.33 for DDX3X [Karczewski et al. 2020]). Both DDX3X and DDX3Y are intolerant of loss-of-function alleles in the gnomAD data set. This indicates both a high degree of conservation of the DDX3 orthologs as well as a potential function and evolutionary flexibility for DDX3Y distinct from its X-linked paralog.

**FIGURE 2.**
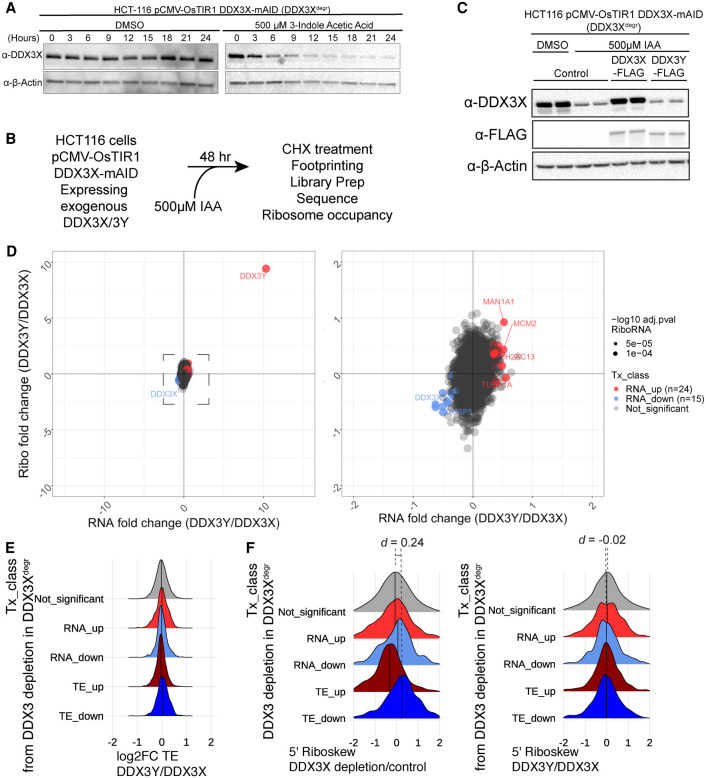
(*A*) Western blot for degradation of endogenous DDX3X tagged with mini-AID (mAID) upon treatment with an auxin (indole-3-acetic acid) in HCT-116 human colorectal carcinoma cell line expressing OsTIR1 under the control of a CMV promoter (DDX3^degr^). (*B*) Experimental schematic for ribosomal profiling after replacement of endogenous DDX3X with exogenous DDX3X or 3Y. (*C*) Western blot for expression of exogenous FLAG-tagged DDX3X and DDX3Y in HCT-116 cells after degradation of endogenous DDX3X. (*D, left*) Full and (*right*) zoomed in plots of differential expression analysis of RNA and ribosome profiling changes upon complementation of DDX3X with either DDX3Y or DDX3X. Point size indicates *P*-value of differential translation (two biological replicates, each condition). (*E*) Fold-change in TE between DDX3Y and DDX3X expression in mRNAs classified based on DDX3 sensitivity (as in Supplemental Fig. S2A). (*F*) The fold-change of the riboskew, or ratio in ribosome occupancy in the 5′ UTR versus the coding sequence under DDX3 depletion (*left*) or the ratio between complementation with DDX3Y or DDX3X (*right*). Effect size (Cliff's delta) between the “Not_significant” and “TE_down” groups is indicated.

In order to dissect the similarities and differences between the functions of DDX3X and DDX3Y, we used a cell line we generated to rapidly and efficiently degrade endogenous DDX3 protein X in human male-derived colorectal cancer HCT 116 cells, which show a stable diploid karyotype (Waldman et al. 1995; Natsume et al. 2016). We tagged DDX3X with a 68 amino acid fragment of the Auxin Inducible Degron (AID) tag termed mini-AID (mAID) in parental cells expressing the auxin-responsive F-box protein, TIR1, from *Oryza sativa* (OsTIR1) (Natsume et al. 2016). Cells treated with the synthetic auxin, Indole-3-acetic acid (IAA), exhibit >90% reduction in DDX3X protein levels by 12 h of treatment (Fig. 2A; Calviello et al. 2021). Depletion of DDX3X (48-h auxin treatment only) affects the translation of a subset of cellular messages (Supplemental Fig. S2A). Transcripts with increased DDX3X sensitivity have low basal translation efficiency and increased GC content and RNA structure within their 5′ leader sequences (Supplemental Fig. S2B). This observation is consistent with previously reported results (Calviello et al. 2021).

Lentiviral transduction with either FLAG-tagged *DDX3X* or *DDX3Y* cDNAs followed by degradation of endogenous DDX3X protein allowed for almost complete replacement of the endogenous DDX3 protein with exogenous DDX3X or DDX3Y (Fig. 2B,C). We measured transcript levels and ribosome occupancy under these experimental conditions using ribosome profiling and RNA-seq. Expression of DDX3Y or DDX3X after endogenous DDX3X depletion shows similar transcript levels and ribosome densities across the transcriptome (Fig. 2D). Introduced DDX3X and DDX3Y proteins were expressed to a similar degree (Fig. 2C), but DDX3Y appeared to be more highly expressed at the RNA level (Fig. 2D) because its endogenous expression is low in HCT 116 cells (endogenous DDX3X TPM = 162; endogenous DDX3Y TPM = 0.1; transduced DDX3X TPM = 284; transduced DDX3Y TPM = 174; see Supplemental Table S3). We found that exogenous expression of either DDX3X or DDX3Y rescued almost all changes in translation suffered due to depletion of endogenous DDX3X (99% in DDX3X and 94% in DDX3Y) (Supplemental Fig. S2C, compare to Supplemental Figs. S2A, S3A).

To increase statistical power to detect a difference between DDX3X and DDX3Y, we tested for differences between groups of transcripts. Transcripts sensitive to depletion of DDX3X (as in Supplemental Fig. S2A) show no change in their translation efficiency upon substitution of DDX3X with DDX3Y (Fig. 2E). Additionally, DDX3X-sensitive transcripts show increased ribosome occupancy in 5′ leaders compared to the CDS (5′ riboskew), consistent with previous observations (Fig. 2F; Calviello et al. 2021). However, DDX3X-sensitive transcripts show no change in 5′ riboskew upon the expression of DDX3Y compared to DDX3X (Fig. 2F). Furthermore, transcripts with the largest magnitude of changes in TE between DDX3Y and DDX3X expression show no propensity for increased 5′ UTR GC content, a signature of DDX3 sensitivity (Supplemental Fig. S2D). The small minority of transcripts whose translation was incompletely rescued by exogenous DDX3 paralog expression also show no change in 5′ riboskew—a characteristic of DDX3-sensitive transcripts (Supplemental Fig. S2E). These observations are consistent with the X- and Y-linked paralogs of DDX3 performing redundant functions in translation.

**FIGURE 3.**
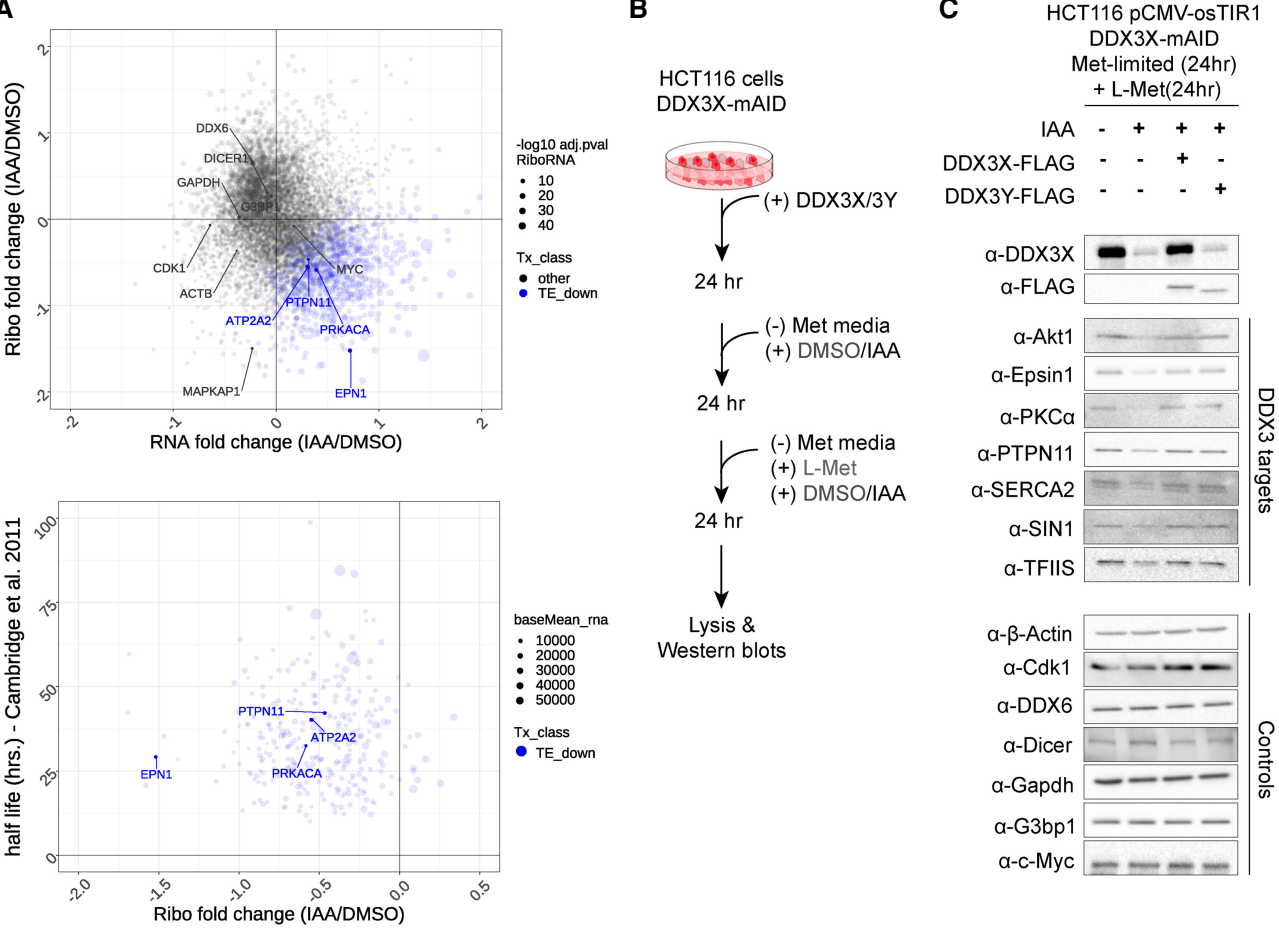
(*A*) Selected DDX3-sensitive transcripts are indicated by the effect of DDX3 on their translation (*top*) and the half-lives of their protein products (*bottom*). (*B*) Experimental schematic for Methionine-chase assay. (*C*) Western blot for protein products of DDX3-sensitive or control transcripts upon DDX3X depletion and complementation with DDX3X or DDX3Y.

We sought to validate the impact of DDX3X depletion on the translation of selected targets and their rescue upon complementation by either DDX3X or DDX3Y. We selected candidates for DDX3 susceptibility (*DVL2* long and short isoforms, *ODC1*, and *PRKRA*) and controls (*ATF5*, *CCNE1*, *RPLP1*, and *SIKE1*) based on a combination of their susceptibility to DDX3 depletion in this and previous studies (Fig. 3A; Supplemental Fig. S3B), previously measured half-lives and availability of antibodies. Ribosome profiling data provide a snapshot of ribosome engagement, and acute changes in ribosome occupancy may or may not result in changes in steady-state protein levels. Therefore, we starved cells of methionine for 24 h to impede translation and allow for clearance of a fraction of previously synthesized proteins by the cellular degradation machinery. We followed by reintroducing complete media to restart protein synthesis and measured protein levels of our candidates by western blot (Fig. 3B). We observe that the protein levels of DDX3 target genes, but not control genes, are reduced upon depletion of DDX3X, and restored upon complementation with either DDX3X or DDX3Y (Fig. 3C).

**FIGURE 4.**
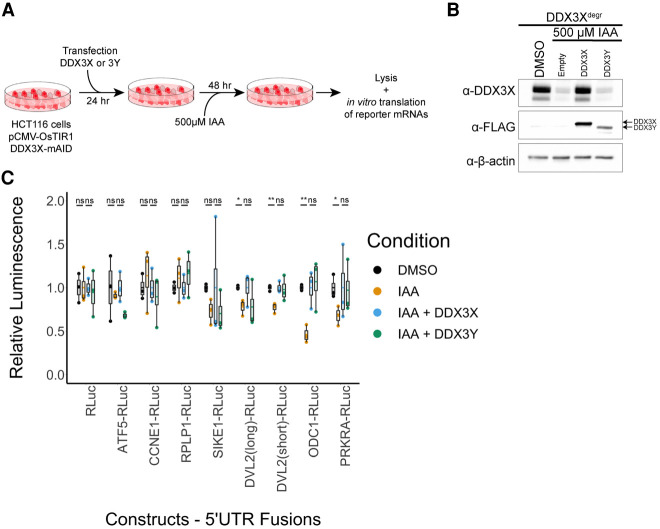
(*A*) Experimental schematic for in vitro translation after replacement of endogenous DDX3X with exogenous DDX3X or 3Y. (*B*) Western blot for degradation of endogenous DDX3X tagged with mini-AID (mAID) and expression of exogenous FLAG-tagged DDX3X and DDX3Y in HCT-116 cells after degradation of endogenous DDX3X in in vitro translation extracts. (*C*) Translation of in vitro transcribed reporter RNAs in lysates from control (DMSO) or DDX3X-degraded (auxin treated) HCT-116 cells as well as either FLAG-tagged DDX3X or DDX3Y on top of depletion of endogenous DDX3X (three biological replicates, each condition).

In order to further test the impact of DDX3X and DDX3Y on translation, we used a set of previously described DDX3-sensitive 5′ UTRs upstream of a Renilla luciferase reporter and performed in vitro transcription, capping and 2′-*O*-methylation to generate a set of reporter RNAs (Calviello et al. 2021). We made translation extracts from HCT 116 cells either depleted of endogenous DDX3X or depleted and complemented by transient transfection of exogenous *DDX3X* and *DDX3Y* (Fig. 4A,B). As previously observed, 5′ UTRs of DDX3-sensitive mRNAs also confer DDX3 dependence to a luciferase reporter, while the control RNA with a simple 5′ UTR remains unaffected (Fig. 4C). Consistent with the ribosome profiling results, DDX3-sensitive 5′ UTRs show no statistically significant change in reporter output when translated with extracts containing only DDX3Y compared to DDX3X (Fig. 4C). This includes the *ODC1* reporter, which is sensitive to every perturbation in DDX3X that we have tested, included depletion and inactivating mutants (Calviello et al. 2021). However, we note that the *DVL2*(long) reporter has a visual difference between DDX3X and DDX3Y complementation that did not reach statistical significance. These data further reinforce the notion that DDX3X and DDX3Y are functionally redundant in regulation of translation.

**FIGURE 5.**
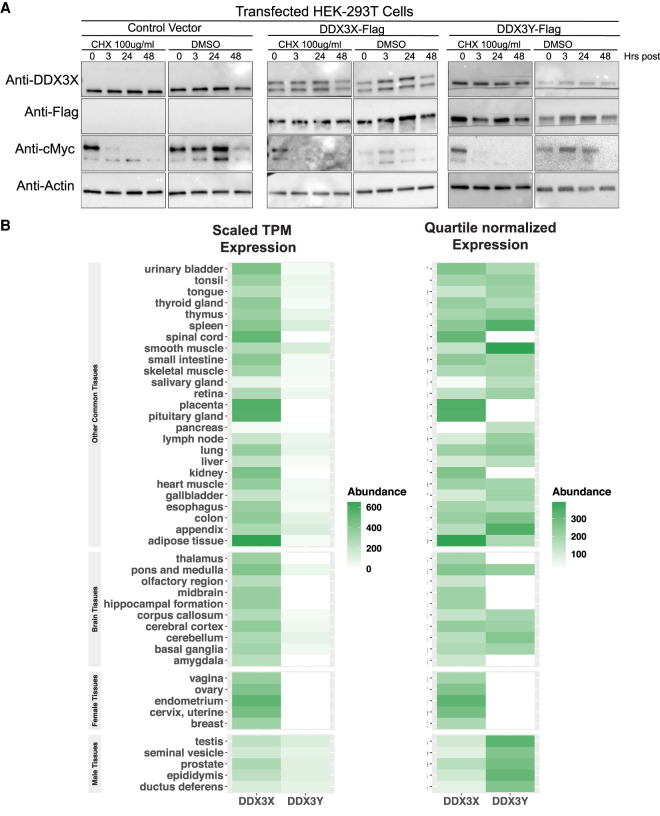
(*A*) Western blots of endogenous and transfected DDX3 paralogs (DDX3X and DDX3Y) for 48 h after treatment with cycloheximide (100 µg/mL). Myc is used as a control for rapid degradation. (*B, left*) Scaled TPM expression of DDX3X and DDX3Y across a number of human tissues (data from GTeX). (*Right*) Quartile-normalized expression of DDX3X and DDX3Y across a number of human tissues (data from GTeX). DDX3Y expresson is particularly prominent in male-specific tissue types such as prostate and seminal vesicles, in the testes, and notably deplete in numerous tissues of the central nervous system.

The results above suggest that DDX3Y can rescue translational deficiencies caused by loss of DDX3X. To reconcile this with the observed sex-bias in DDX3X-dependent disorders, we considered multiple possibilities. To eliminate any role of differences in relative protein degradation rates, we examined the stability of DDX3X or DDX3Y proteins in HEK293T cells by treatment with cycloheximide (CHX). We observed rapid loss of c-Myc but no measurable degradation of either paralog before the onset of severe CHX toxicity (2 d, Fig. 5A). Therefore, even if DDX3Y is more or less stable than DDX3X on a longer timescale than 2 d, both are still very stable proteins. Next, we examined FANTOM-CAGE expression data for the paralogs across multiple human tissues (Lizio et al. 2015). Our examination confirmed that expression of *DDX3Y* is prominent in the tissues of the male reproductive system (prostate, seminal vesicle, testes etc.; Fig. 5B). Furthermore, *DDX3Y* expression is undetectable in several cell types in the brain, including the midbrain, hippocampus, thalamus and amygdala (Fig. 5B). Taken together, we found that *DDX3X* and *DDX3Y* functioned similarly in translation and are differentially expressed at the RNA level between human tissues.

## DISCUSSION

The DEAD-box protein DDX3 is encoded by two paralogous genes in humans, *DDX3X* and *DDX3Y,* the protein products of which show 92% sequence identity (Fig. 1B). Heterozygous inactivating mutations in *DDX3X* are linked to DDX3X syndrome, a neurodevelopmental delay and autism-spectrum disorder (Lennox et al. 2020). DDX3X syndrome shows a severe sex bias with >95% of known cases affecting females—indicating that *DDX3Y* is unable to compensate for the impairment of *DDX3X* function in brain despite recent observations that *DDX3Y* may also modulate neural development (Vakilian et al. 2015). Conversely, loss of *DDX3Y* results in a male infertility phenotype despite robust expression of DDX3X in male germline tissues, indicating that *DDX3X* cannot complement loss of *DDX3Y* in the male reproductive system (Rauschendorf et al. 2014; Ramathal et al. 2015; Kotov et al. 2017)

In this and previous work, we have demonstrated using orthogonal systems that DDX3X is required for translation initiation on transcripts with structured 5′ leader sequences (Supplemental Fig. S2A,B; Calviello et al. 2021). In this study, we examined the global translation profiles when cells depleted of endogenous DDX3X are complemented with cDNAs of either *DDX3X* or *DDX3Y*. We could not detect significant changes of translation or steady state RNA levels (Fig. 2). We used in vitro translation experiments with a luciferase reporter to confirm 5′ leaders of genes that are sensitive to either depletion of DDX3X or inactivating mutations show no significant differences in translation in extracts containing either DDX3X or DDX3Y (Fig. 4). In fact, the genes that show decreased translation (TE_down) upon depletion of DDX3X show an increased change in the relative ribosome occupancy in the 5′ leader compared to the CDS, a signature that is lost while comparing DDX3Y to DDX3X (Fig. 2; Supplemental Fig. S2). This suggests that the two human paralogs of DDX3 perform equivalent functions in translation, at least in the context of our experimental system.

At least six models could potentially explain the inability of the DDX3 paralogs to complement each other. First, the two paralogs of DDX3 could have distinct target complements and affect the translation of different sets of transcripts. Second, the two paralogs could have the same target propensity but distinct subcellular localizations, and thus differ in their effects. These possibilities are partially rebuffed by the gain of DDX3Y expression complementing for loss of DDX3X in certain lymphoid malignancies (Gong et al. 2021) and in BHK21 hamster cells (Sekiguchi et al. 2004). Additionally, a genome-wide CRISPR screen for essential genes identified *DDX3Y* as being essential only in Raji cells (which are also of hematopoietic origin), which had suffered a truncating mutation in DDX3X, further indicating genetic complementation (Wang et al. 2015). However, genetic complementation might mask a subtle fitness defect or gene expression changes that could be important in other cellular states. Third, the two differences in protein sequences between the paralogs could lead to differential stability and residence time in the cell. In this study, we quantitatively demonstrate that the translation profile of DDX3X and DDX3Y-expressing cells are functionally identical, and that the paralogs are both exceptionally long-lived in cells, arguing against the three scenarios laid out above.

A fourth model to explain the lack of paralog complementation at the organism level is tissue-specific expression patterns of the paralogs. At the RNA level, *DDX3Y* expression is sporadic in the different cell types of the brain, as well as all tissues excepting those of the male reproductive system (Fig. 5). It has previously been suggested that specific transcriptional and translational regulation contribute to increased DDX3Y protein levels in the testes (Jaroszynski et al. 2011; Rauschendorf et al. 2011). One potential explanation for the inability of *DDX3Y* to complement *DDX3X* in the brain could be that the protein is expressed in insufficient quantities. Conversely, although DDX3X is detectable in adult male testes, multiple layers of transcriptional and translational control restrict its expression to post-meiotic spermatids (Rauschendorf et al. 2014). The absence of DDX3X protein in premeiotic spermatids could explain its inability to rescue the infertility phenotype conferred by *DDX3Y* loss. A recent analysis of tissue-specific expression of sex-linked paralogs correcting for multimapping short sequencing reads estimated that while the cumulative expression of DDX3 is statistically identical across tissues for individuals with XX and XY genotypes, the DDX3Y expression makes up only a small minority of total DDX3 (Godfrey et al. 2020). This lends further support to the idea that while the two paralogs are functionally identical, spatiotemporal differences in their expression precludes complementation in a number of scenarios. It is important to note that this model requires future work to be confirmed. Lineage-specific complementation of DDX3 paralogs in vivo would formally test this hypothesis, and there are already hints of paralog-specific developmental effects (Patmore et al. 2020).

Two models remain that cannot be formally ruled out. First, there could be context-dependent factors in neurodevelopment and germ-cell development that cause differential translational regulation by the X- and Y- linked DDX3 paralogs, and that these contribute to their inability to complement each other in these contexts—despite the gene products of *DDX3X* and *DDX3Y* having redundant functions. Most of the differences in the polypeptide sequences of DDX3X and DDX3Y are in the amino- and carboxy-terminal domains, outside the helicase core, that are sites of protein–protein interactions. It is conceivable that these differences might drive differences in the interactomes of the paralogs. Some evidence in support of this model comes from the observation that in mice, while *Ddx3y* is able to compensate for the neurodevelopmental phenotypes caused by the loss of *Ddx3x* in neural lineages, it is unable to suppress the increase in hindbrain malignancies also caused by *Ddx3x* loss (Patmore et al. 2020). This would indicate some form of context-dependent accessory factor influencing the target complement of Ddx3 homologs. However, it is worth noting that the ability of male mice with genetic lesions in *Ddx3x* to survive without neurological anomalies has not been recapitulated in humans as genetic variation in *DDX3X* in the human population is extremely low (Karczewski et al. 2020). Lastly, it is formally possible that there are mechanisms outside protein synthesis that both DDX3X and DDX3Y contribute to and that they function differently in those other functions. This work focused on the role of DDX3X and DDX3Y in protein synthesis, where data show they function redundantly, but the marginal differential expression that persists after complementation could be the result of functions outside protein synthesis (Supplemental Fig. S2C).

We suggest that some combination of tissue-specific paralog and unknown cofactor expression may explain the sex bias of DDX3X-associated developmental disorders, but that inherent differences in molecular function are unlikely to drive this phenomenon. Further investigations in these directions could reveal specific targets or factors that could be exploited to mitigate the phenotypic consequences of genetic lesions in the *DDX3* paralogs.

## MATERIALS AND METHODS

### DDX3 sequence alignment and phylogenetic tree

Reference protein sequences of human DDX3X and DDX3Y were obtained from RefSeq, and alignment was performed using the MAFFT algorithm using the BLOSUM62 scoring matrix with default parameters (gap open penalty = 1.53; gap extension penalty = 0.123) (Katoh et al. 2002). Protein sequences for mammalian DDX3 orthologs (both X and Y, where available and reliably annotated) were obtained using a combination of the NCBI Gene ortholog finder and PSI-blast (Altschul et al. 1997). Multiple sequence alignment was performed as described above, and a phylogenetic tree was constructed using the Interactive Tree of Life (iTOL) tool (v5.6.3) (Letunic and Bork 2019).

### Cell line construction

HCT-116 cells expressing OsTIR1 were constructed as described in Natsume et al. (2016). Briefly, a plasmid coexpressing gRNA for the AAVS1 safe-harbor locus and SpCas9 was cotransfected with plasmid encoding for OsTIR1 and homologous arms for the HDR at the AAVS1 locus. Selection was performed in 1 µg/mL of puromycin; colonies were picked and validated by PCR and western blot.

The HCT-116 OsTIR1 expressing cell line was transfected with 800 ng (total) of an equimolar mixture of pX330-based CRISPR/Cas plasmid with guide RNAs targeting the carboxy-terminal end of DDX3X and 1200 ng of a short homology donor carrying the mAID degron tag with a G418 resistance cassette in a pUC19 backbone. Selection was performed in 700 µg/mL of G418, and single colonies picked and validated by PCR and western blot. Selection, picking, clonal expansion, and validation were repeated twice (total of three rounds) to ensure a clonal cell line.

Lentiviral constructs expressing FLAG-tagged and partially codon-optimized DDX3X or DDX3Y (ORFs obtained from Twist Biosciences, cloned upstream of tandem P2A cleavage site followed by mCherry2) were transfected into HEK293T cells along with packaging plasmids. Three days post transfection, the media containing viral particles were harvested and filtered, before being used for infection of the DDX3X-degron-containing cell line described above. Cells with successful expression of DDX3 paralogs were isolated by sorting for mCherry2 positive cells. Three rounds of sorting were performed to ensure stable expression. Levels of DDX3X or DDX3Y protein were confirmed by western blot against the FLAG epitope.

### Induction of DDX3X degradation

For ribosome profiling, 500 µM Indole-3-acetic acid (IAA, Grainger, 31FY95) was added to the cells (in fresh media) from a 500 mM stock in DMSO. For in vitro translation lysates, CMV-promoter expression plasmids (50 µg of DDX3X-FLAG and 75 µg of DDX3Y-FLAG per 15 cm plate, to equalize expression levels) were transfected into cells using a 3:1 ratio of lipofectamine 2000 to plasmid. Twenty-four hours post-transfection, 500 µM Indole-3-acetic acid (IAA, Grainger, 31FY95) was added to the cells (in fresh media) from a 500 mM stock in DMSO. Protein levels were assayed by western blot.

### Antibodies

Primary antibodies used in this study include rabbit polyclonal anti-DDX3 (custom made by Genemed Synthesis using peptide ENALGLDQQFAGLDLNSSDNQS) (Lee et al. 2008), anti-actin HRP (Santa Cruz, sc-47778), anti-FLAG HRP (Sigma, A8592), anti-Akt1 (Bethyl, A302-065A), anti-Epsin1 (Bethyl, A304-524A), anti-PKCα (Bethyl, A302-445A), anti-PTPN11 (Bethyl, A301-544A), anti-SERCA2 (Bethyl, A300-406A), anti-Sin1 (Bethyl, A300-910A), anti-TFIIS (Bethyl, A302-240A), anti-Cdk1 (Santa Cruz, sc-54), anti-DDX6 (Novus, NB200-191), anti-Dicer (SantaCruz, sc-136979), anti-GAPDH, anti-G3BP1 (Bethyl A302-034A), and anti-cMyc (abcam, ab205818).

### Ribosome profiling library construction

One 15 cm dish of cells at 80%–90% confluency was used per replicate. Twenty-four hours post plating, media were changed and fresh media with 500 µM IAA were added to cells. Forty-eight hours after auxin addition, cells were treated with 100 µg/mL cycloheximide (CHX) and harvested and lysed as described in McGlincy and Ingolia (2017). Briefly, cells were washed with PBS containing 100 µg/mL CHX and lysed in ice-cold lysis buffer (20 mM TRIS-HCl pH 7.4, 150 mM NaCl, 5 mM MgCl_2_, 1 mM DTT, 100 µg/mL CHX, 1% [v/v] Triton X-100, 25 U/mL TurboDNase [Ambion]). A total of 240 µL lysate was treated with 6 µL RNase I (Ambion, 100 U/µL) for 45 min at RT with gentle agitation and further digestion halted by addition of SUPERase•In (Ambion). Illustra Microspin Columns S-400 HR (GE Healthcare) were used to enrich for monosomes, and RNA was extracted from the flow-through using a Direct-zol Kit (Zymo Research). Gel slices of nucleic acids between 24–32 nt long were excised from a 15% urea-PAGE gel. Eluted RNA was treated with T4 PNK and preadenylated linker was ligated to the 3′ end using T4 RNA Ligase 2 truncated KQ (NEB, M0373L). Linker-ligated footprints were reverse transcribed using Superscript III (Invitrogen) and gel-purified RT products circularized using CircLigase II (Lucigen, CL4115K). rRNA depletion was performed using biotinylated oligos as described in Ingolia et al. (2012) and libraries constructed using a different reverse indexing primer for each sample.

RNA was extracted from 25 µL intact lysate (nondigested) using the Direct-zol Kit (Zymo Research), and stranded total RNA libraries were prepared using the TruSeq Stranded Total RNA Human/Mouse/Rat Kit (Illumina), following manufacturer's instructions.

Libraries were quantified and checked for quality using a Qubit fluorimeter and Bioanalyzer (Agilent) and sequenced on a HiSeq 4000 sequencing system (single end, 65 nt reads).

### Preprocessing and alignment of NGS data

All NGS data were processed as previously described (Calviello et al. 2021). Briefly, adapter sequences were trimmed from the footprint reads, UMI sequences were collapsed and removed. Reads aligned to rRNA or a collection of snoRNAs, tRNAs, and miRNAs were discarded. The filtered reads were mapped to the hg38 version of the human genome using STAR (Dobin et al. 2013) and count matrices built as described previously (Calviello et al. 2021) using Ribo-seQC (Calviello et al. 2019). Only reads above 24 nt long were counted. Read counts for all libraries are in Supplemental Table S1.

### Differential expression analysis

Differentially expressed (DE) genes between the samples were identified using DESeq2 (Love et al. 2014). Only genes with average normalized number of counts greater than 20 for both RNA and Ribo-seq were retained. Genes with transcript levels were defined as either “RNA_up” or “RNA_down,” with an adjusted *P*-value cutoff of 0.01. Differential translation regulation was calculated using DESeq2 interaction terms and a likelihood ratio test, with an adjusted *P*-value cutoff of 0.05, and differentially translated genes were defined as “TE-up” or “TE_down.” DE details for all genes that met the read count cutoff are in Supplemental Table S2.

### Transcript features analysis

5′UTR ribosome skew (riboskew) was calculated as previously defined (Calviello et al. 2021). Effect sizes (nonparametric Cliff's Delta measure) between the transcript classes were calculated using the “effsize” R package (Torchiano 2020).

### Methionine starvation and chase

Candidates for validation of ribosome profiling were chosen based on effects of DDX3X depletion of translation efficiency, measured half-lives (Cambridge et al. 2011), and availability of antibodies. Expression plasmids carrying DDX3X-FLAG or DDX3Y-FLAG were transfected into cells using a 3:1 ratio of lipofectamine 2000 to plasmid. Twenty-four hours post-transfection, cells were washed three times with PBS to eliminate residual methionine. A total of 500 µM Indole-3-acetic acid (IAA, Grainger, 31FY95) was added to the cells in fresh media lacking methionine from a 500 mM stock in DMSO. Cells were maintained in methionine deficient media for 24 h, following which media were replaced with complete media (containing IAA or DMSO). Cells were harvested 24 h later and lysed for western blot analysis.

### In vitro transcription, capping, and 2′-*O*-methylation of reporter RNAs

Annotated 5′ UTRs for selected transcripts were cloned upstream of Renilla Luciferase (RLuc) under the control of a T7 promoter, with 60 adenosine nucleotides downstream from the stop codon to mimic polyadenylation. Untranslated regions were cloned using synthetic DNA (Integrated DNA Technologies) or by isolation using 5′ RACE (RLM-RACE Kit, Invitrogen). Template was PCR amplified using Phusion polymerase from the plasmids using the following primers, and gel purified, as described (Floor and Doudna 2016).

pA60 txn rev: TTT TTT TTT TTT TTT TTT TTT TTT TTT TTT TTT TTT TTT TTT TTT TTT TTT TTT TTT TTT CTG CAG
pA60 txn fwd: CGG CCA GTG AAT TCG AGC TCT AAT ACG ACT CAC TAT AGG

A total of 100 µL in vitro transcription reactions were set up at room temperature with 1–5 µg of purified template, 7.5 mM ACGU ribonucleotides, 30 mM Tris-Cl pH 8.1, 125 mM MgCl_2_, 0.01% Triton X-100, 2 mM spermidine, 110 mM DTT, T7 polymerase, and 0.2 U/µL of Superase•In RNase inhibitor (Thermo Fisher Scientific). Transcription reactions were incubated in a PCR block at 37°C for 1 h. A total of 1 µL of 1 mg/mL pyrophosphatase (Roche) was added to each reaction, and the reactions were subsequently incubated in a PCR block at 37°C for 3 h. One unit of RQ1 RNase-free DNase (Promega) was added to each reaction followed by further incubation for 30 min. RNA was precipitated by the addition of 200 µL 0.3 M NaOAc pH 5.3, 15 µg GlycoBlue coprecipitant (Thermo Fisher Scientific), and 750 µL 100% EtOH. Precipitated RNA was further purified over the RNA Clean & Concentrator-25 columns (Zymo Research). Glyoxal gel was run to assess the integrity of the RNA before subsequent capping and 2′-*O*-methylation.

An amount of 20 µg of total RNA was used in a 40 µL capping reaction with 0.5 mM GTP, 0.2 mM S-adenosylmethionine (SAM), 20 units of Vaccinia capping enzyme (New England Biolabs), 100 units of 2′-*O*-methyltransferase (New England Biolabs), and 25 units RNasin Plus RNase Inhibitor (Promega). The reactions were incubated at 37°C for 1 h, followed by purification over the RNA Clean & Concentrator-25 columns (Zymo Research) and elution in DEPC H2O. Glyoxal gel was run to assess the integrity of the RNA before proceeding to in vitro translation reactions.

### Preparation of cellular extracts for in vitro translation

Three 150 mm plates of HCT 116 cells were trypsinized and pelleted at 1000*g*, 4°C. One cell-pellet volume of lysis buffer (10 mM HEPES, pH 7.5, 10 mM KOAc, 0.5 mM MgOAc_2_, 5 mM DTT, and one tablet miniComplete EDTA free protease inhibitor [Roche] per 10 mL) was added to the cell pellet and was incubated on ice for 45 min. The pellet was homogenized by trituration through a 26G needle attached to a 1 mL syringe 13–15 times. Efficiency of disruption was checked by trypan blue staining (>95% disruption target). The lysate was cleared by centrifugation at 14,000*g* for 1 min at 4°C, 2–5 µL was reserved for western blot analysis, and the remainder was aliquoted and flash frozen in liquid nitrogen. Preparation of HEK293T lysates with DDX3X siRNA knockdown and mutant expression has been previously described (Lennox et al. 2020)

### In vitro translation

A total of 5 µL in vitro translation reactions were set up with 2.5 µL of lysate and 20 ng total RNA (0.84 mM ATP, 0.21 mM GTP, 21 mM Creatine Phosphate, 0.009 units/mL Creatine phosphokinase, 10 mM HEPES pH 7.5, 2 mM DTT, 2 mM MgOAc, 100 mM KOAc, 0.008 mM amino acids, 0.25 mM spermidine, 5 units RNasin Plus RNase inhibitor [Promega]) as described by Lee et al. (2015). Reaction tubes were incubated at 30°C for 45 min, and expression of the reporter was measured using the Renilla Luciferase Assay System (Promega) on a GloMax Explorer Plate Reader (Promega).

### Analysis of DDX3 paralog stability

One 10 cm dish of HEK-293T cells at ∼80% confluency was used per replicate. Twenty-four hours post plating, cells were transfected with a DDX3X-FLAG, DDX3Y-FLAG, or empty vector using the TransIT-LT1 Transfection Reagent System (Mirus). Plates were split into a 12 well plate 24 h post transfection. Twenty-four hours after resting, cells were collected for a 0 h sample. The remaining cells were treated with either 100 µg/mL cycloheximide (CHX) or DMSO and collected and flash frozen in liquid nitrogen 3, 24, and 48 h post treatment. Samples were thawed, lysed in buffer (150 mM NaCl, 1% IGEPAL, 50 mM Tris-HCl pH 8.0), homogenized, and centrifuged. The supernatant was saved for western blot analysis.

### Analysis of FANTOM5 tissue level expression data

FANTOM5 per gene transcript expression levels (CAGE data) was obtained based on The Human Protein Atlas version 19.3 and Ensembl version 92.38 (Lizio et al. 2015). Quantile-normalized and scaled TPMs were plotted for common tissues as well as male and female-specific tissues.

## DATA DEPOSITION

Sequencing data can be retrieved using GEO accession number GSE180669. Source code for the analysis of ribosome profiling data was adapted from https://github.com/lcalviell/DDX3X_RPCLIP. Source code specific to the analysis in this manuscript is available at https://github.com/srivats-venkat/DDX3X_3Y_comparison.

## SUPPLEMENTAL MATERIAL

Supplemental material is available for this article.

## COMPETING INTEREST STATEMENT

S.N.F. consults for MOMA Therapeutics.

## Supplementary Material

Supplemental Material
