# Supplementary material for: DDX3X and DDX3Y are redundant in protein synthesis
Source: RNA. 2021 Dec;27(12):1577–88. doi: 10.1261/rna.078926.121 (PMC8594478; doi:10.1261/rna.078926.121)
Supplement: Supplemental Material [file supp_27_12_1577__DC1.html]

DDX3X and DDX3Y are redundant in protein synthesis — DDX3X And DDX3Y are redundant in protein synthesis — Supplemental Material 

# DDX3X and DDX3Y are redundant in protein synthesis

## Supplemental Material

- Supplemental\_FigureS1.pdf
- Supplemental\_FigureS2.pdf
- Supplemental\_FigureS3.pdf
- Supplemental\_Table\_S1.tsv
- Supplemental\_Table\_S2.xlsx
- Supplemental\_Table\_S3.xlsx
