# Supplementary material for: DDX3X and DDX3Y are redundant in protein synthesis
Source: RNA. 2021 Dec;27(12):1577–88. doi: 10.1261/rna.078926.121 (PMC8594478; doi:10.1261/rna.078926.121)
Supplement: Supplemental Material [file supp_078926.121_Supplemental_FigureS1.pdf]

Figure S1 (related to Figure 1)

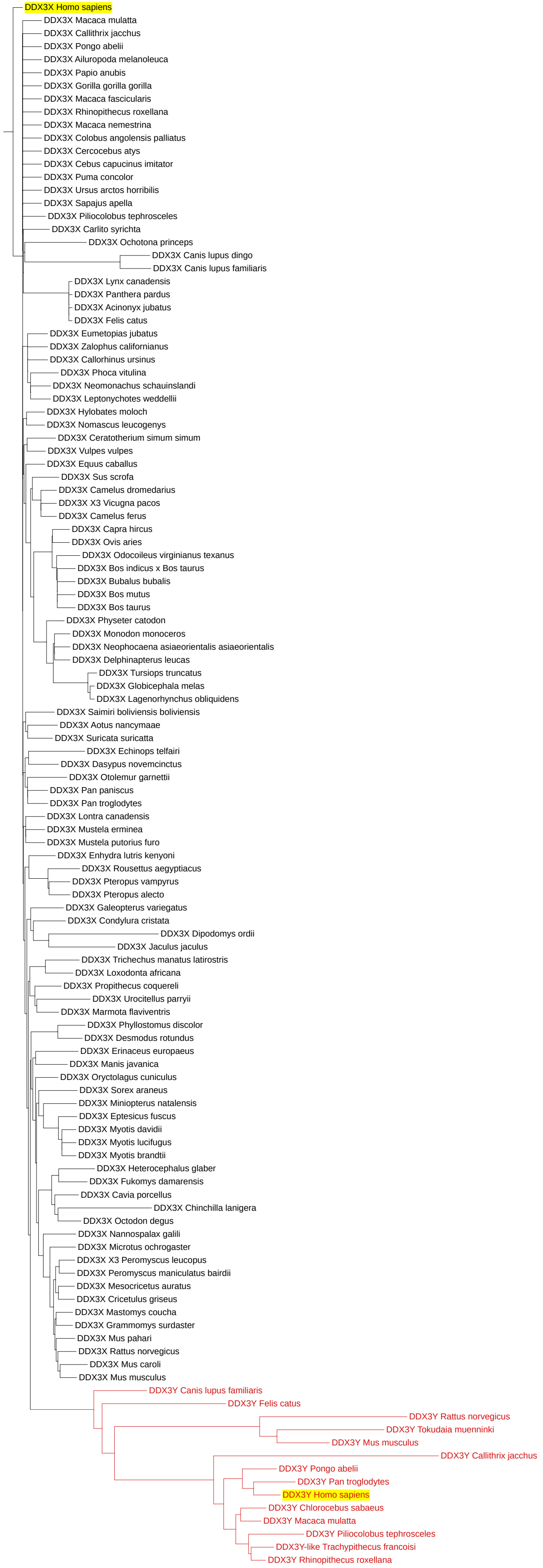

Figure S1: Phylogenetic tree indicating distances between the sequences of DDX3X and DDX3Y in mammals (where sequence is available). Human DDX3X and DDX3Y are highlighted in yellow. The cluster of mammalian DDX3Y orthologs is indicated by red branches.
