# Supplementary material for: DDX3X and DDX3Y are redundant in protein synthesis
Source: RNA. 2021 Dec;27(12):1577–88. doi: 10.1261/rna.078926.121 (PMC8594478; doi:10.1261/rna.078926.121)
Supplement: Supplemental Material [file supp_078926.121_Supplemental_FigureS2.pdf]

Figure S2 (related to Figure 2)

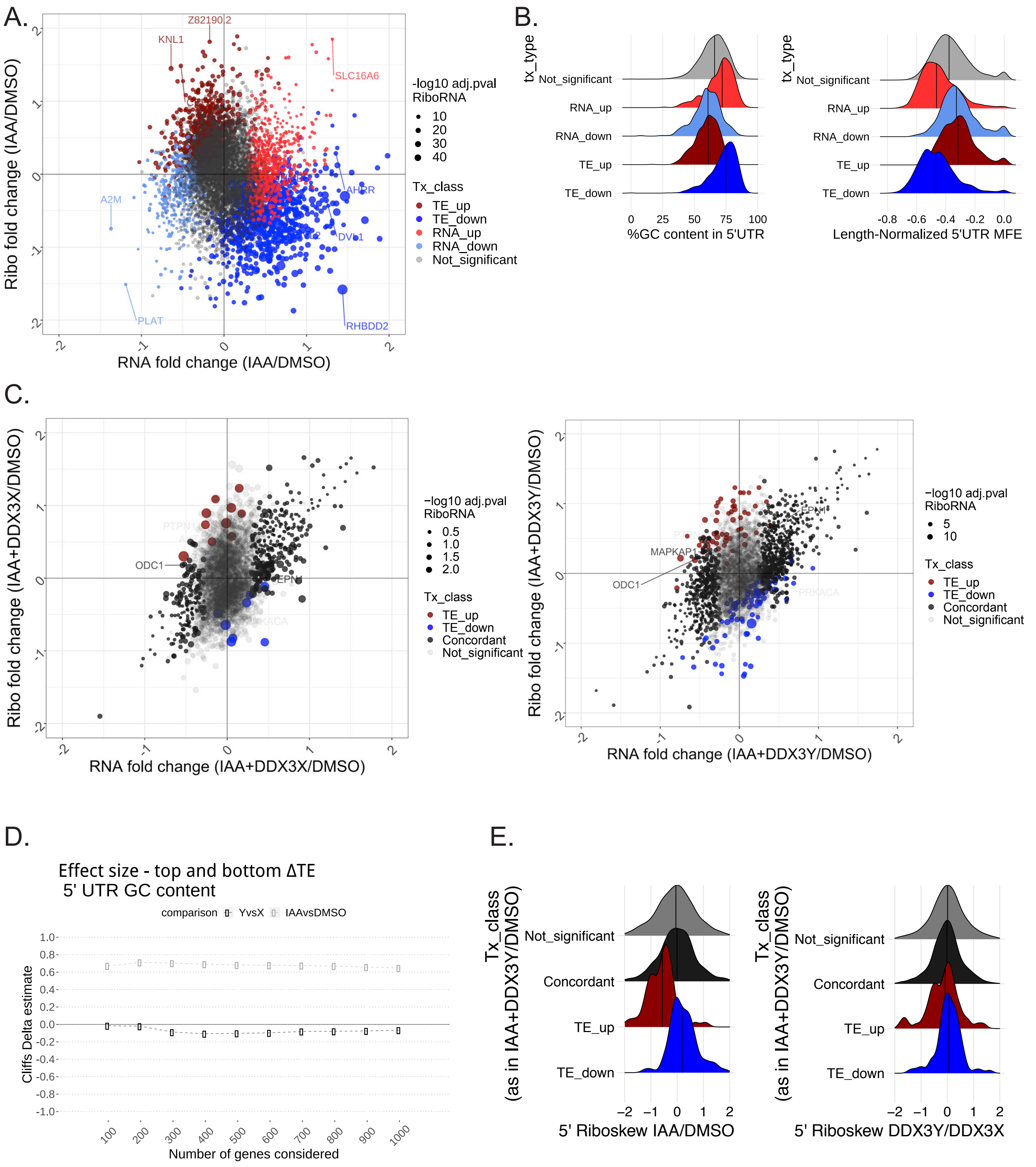

Figure S2:A. Differential expression analysis of RNA and ribosome profiling changes upon DDX3 degradation (two biological replicates, each condition). Point size indicates differential translation **B.** GC content and lenght-normalized mean free energy (MFE) in the 5′ UTR of transcripts in the classes defined in panel A. **C.** Differential expression analysis of RNA and ribosome profiling changes upon complementation of endogenous DDX3X with either DDX3X (left) or DDX3Y (Right) compared to endogenous DDX3X (degron cell line treated with DMSO vehicle). Point size indicates p-value of differential translation **D.** Effect size (Cliff's delta) between 5'UTR GC content of gene sets with indicated Ns with the greatest magnitude of increase (TE_up) or decrease (TE_down) in translation efficiency upon expression of DDX3Y vs. DDX3X or IAA vs. DMSO (without accounting for statistical significance). **E.** The fold-change of the riboskew, or ratio in ribosome occupancy in the 5′ UTR versus the coding sequence in genes classified as in Figure S2C-right under DDX3 depletion (**left**) or the ratio between complementation with DDX3Y or DDX3X (**right**).
