# Supplementary material for: DDX3X and DDX3Y are redundant in protein synthesis
Source: RNA. 2021 Dec;27(12):1577–88. doi: 10.1261/rna.078926.121 (PMC8594478; doi:10.1261/rna.078926.121)
Supplement: Supplemental Material [file supp_078926.121_Supplemental_FigureS3.pdf]

Figure S3 (related to Figure 3)

A.

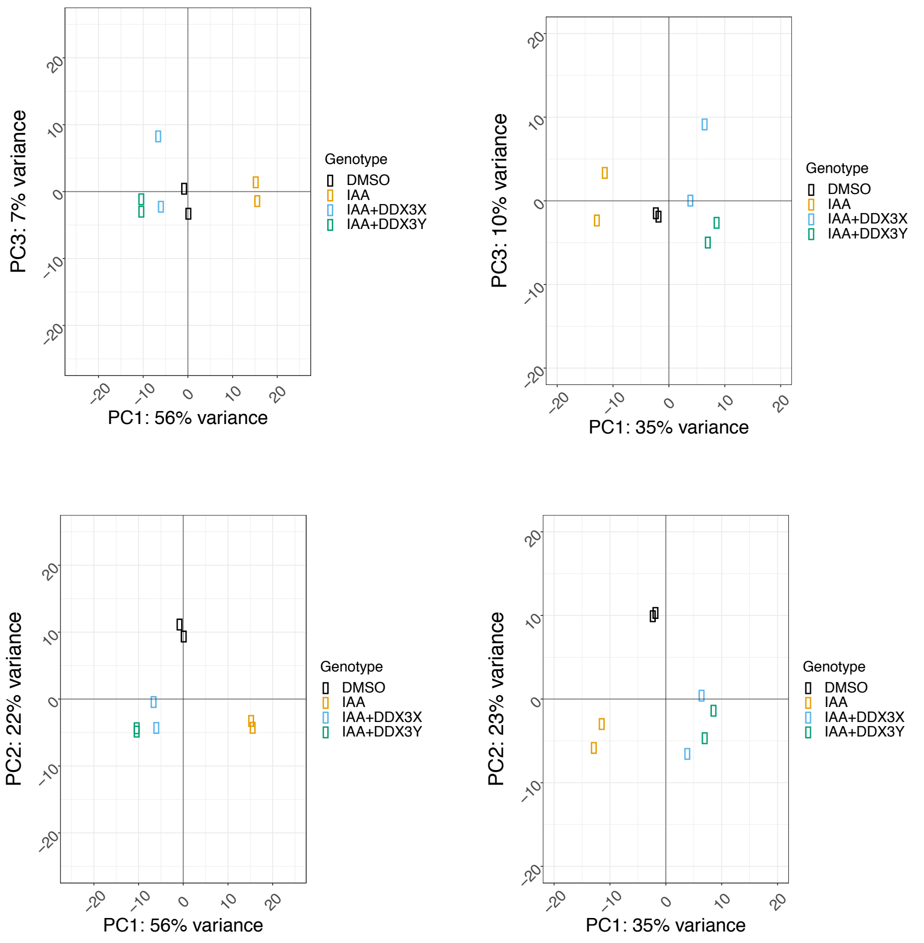

B.

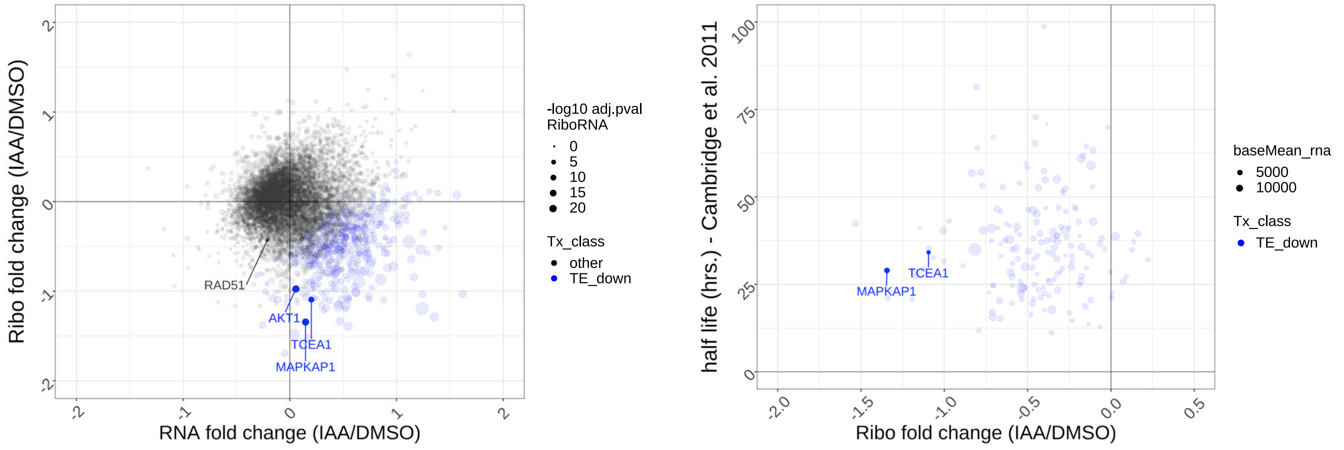

Figure S3:A. Principal component plots of **Left:** RNA levels and **Right:** ribosome occupancy in HCT116 cells transfected with DDX3 variants. Top row depicts PC1 and PC3, indicating the complementation of endogenous DDX3X depletion by wither exogenous DDX3X or DDX3Y. Bottom row depicts PC2 (which correlates with auxin treatment). **B.** Selected DDX3 sensitive transcripts are indicated by the effect of DDX3 on their translation (top, data from Calviello, Venkataramanan et al. 2021) and the half-lives of their protein products (bottom)
